# Inflammatory Cell Dynamics after Murine Femoral Artery Wire Injury: A Multi-Parameter Flow Cytometry-Based Analysis

**DOI:** 10.3390/cells12050689

**Published:** 2023-02-22

**Authors:** Vivek Pamulapati, Carla M. Cuda, Tracy L. Smith, Jonathan Jung, Liqun Xiong, Suchitra Swaminathan, Karen J. Ho

**Affiliations:** 1 Division of Vascular Surgery, Feinberg School of Medicine, Northwestern University, Chicago, IL 60611, USA; 2 Division of Rheumatology, Feinberg School of Medicine, Northwestern University, Chicago, IL 60611, USA

**Keywords:** mice, femoral artery, inflammation, hyperplasia, angioplasty, leukocyte count, flow cytometry, macrophages, neutrophils, monocytes, eosinophils, dendritic cells

## Abstract

An acute inflammatory response following arterial surgery for atherosclerosis, such as balloon angioplasty, stenting, and surgical bypass, is an important driver of neointimal hyperplasia after arterial injury, which leads to recurrent ischemia. However, a comprehensive understanding of the dynamics of the inflammatory infiltrate in the remodeling artery is difficult to attain due to the shortcomings of conventional methods such as immunofluorescence. We developed a 15-parameter flow cytometry method to quantitate leukocytes and 13 leukocyte subtypes in murine arteries at 4 time points after femoral artery wire injury. Live leukocyte numbers peaked at 7 days, which preceded the peak neointimal hyperplasia lesion at 28 days. Neutrophils were the most abundant early infiltrate, followed by monocytes and macrophages. Eosinophils were elevated after 1 day, while natural killer and dendritic cells gradually infiltrated over the first 7 days; all decreased between 7 and 14 days. Lymphocytes began accumulating at 3 days and peaked at 7 days. Immunofluorescence of arterial sections demonstrated similar temporal trends of CD45^+^ and F4/80^+^ cells. This method allows for the simultaneous quantitation of multiple leukocyte subtypes from small tissue samples of injured murine arteries and identifies the CD64^+^Tim4^+^ macrophage phenotype as being potentially important in the first 7 days post-injury.

## 1. Introduction

Surgical interventions for atherosclerosis, such as balloon angioplasty, bypass grafting, and stenting, are considered a form of acute arterial “injury.” Transluminal endothelial disruption exposes the underlying extracellular matrix, leading to rapid deposition of platelets, coagulation proteins, and leukocytes. The ensuing thrombo-inflammatory response is characterized by leukocyte recruitment, the elaboration of cytokines, and vascular smooth muscle cell activation and phenotype switch, which can lead to the subsequent development of neointimal hyperplasia, a common cause of restenosis and recurrent ischemia requiring reoperation or other interventions [1,2,3,4,5,6,7,8,9]. In humans, the extent of neointimal formation correlates with inflammation [10,11,12,13], and there is preclinical evidence that modulation of inflammation mitigates neointimal hyperplasia following vascular surgery [14,15,16]. Characterizations of the post-injury cellular inflammatory response have commonly relied on the quantitation of cell markers on individual arterial sections by immunofluorescence or immunohistochemistry [4,8,17,18,19,20,21,22,23,24]. While this allows for spatial localization of the cellular infiltrate in the arterial wall, it is time-consuming, labor-intensive, susceptible to bias, and does not provide global quantitation of the cellular infiltrate along the length of the injured artery.

Murine models of neointimal hyperplasia after arterial injury include carotid ligation [25], femoral artery wire injury [26], carotid artery wire injury [27], and placement of an external cuff [28]. Of all these models, femoral artery wire injury most closely phenocopies balloon angioplasty in humans since it utilizes transluminal endothelial denudation and enlargement of the external elastic lamina, which elicits fibrin deposition, platelet accumulation, and infiltration of inflammatory cells [29]. By 4 weeks after injury, there is reproducible luminal narrowing due to a neointimal lesion composed of smooth muscle cells [29].

While traditional flow cytometry panels were limited to 3–4 colors, multi-parameter instruments are capable of measuring 12 or more colors [30] in small sample sizes. Herein, we describe a multi-parameter panel, validated by immunofluorescence studies, to obtain details and insights into the dynamics of the inflammatory cellular infiltrate after murine femoral artery wire injury.

## 2. Materials and Methods

### 2.1. Unilateral Femoral Artery Wire Injury

**Figure 1:**
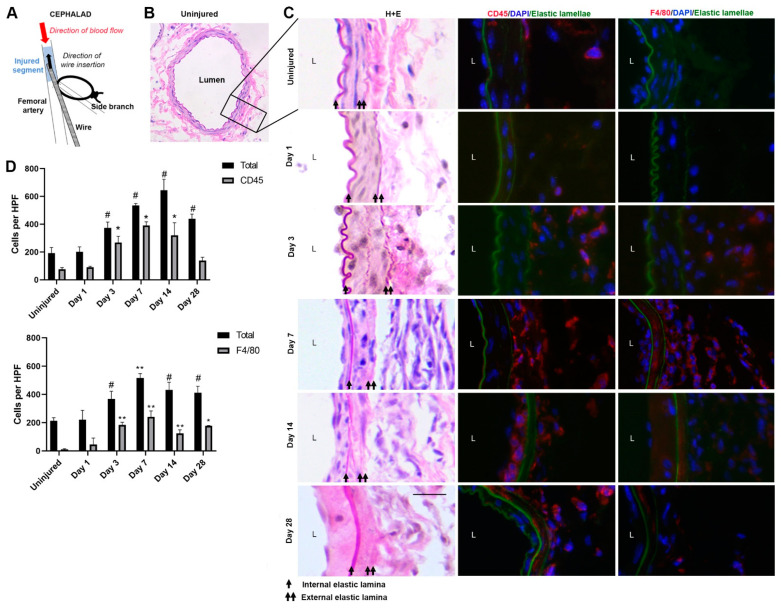
A. Schematic of mouse femoral artery wire injury model. (**A**) guidewire is inserted into the lumen of the artery through a muscular side branch and used to denude the endothelium and dilate the artery. Following removal of the wire, the branch is ligated, and the artery is reperfused. The injured segment is denoted in blue. (**B**) Representative hematoxylin and eosin (H + E) staining of the full cross-section of an uninjured artery. (**C**) Representative H + E and immunofluorescence for CD45 and F4/80 in uninjured (inset of B) and injured femoral artery cross sections 1, 3, 7, 14, and 28 days after injury. Internal and external lamellae are denoted by black arrows on H + E sections. “L” denotes arterial lumen. Red denotes CD45 or F4/80 staining. Green represents autofluorescence of the elastic lamellae. Original magnification 200×. Scale bar represents 5 µM. (**D**) Quantification of CD45^+^ (gray bars in top plot) and F4/80^+^ cells (gray bars in bottom plot) by immunofluorescence at 1, 3, 7, 14, and 28 days after injury and in corresponding uninjured arteries from the same animals. Total cell number (black bars) was determined by DAPI-stained nuclei on the same sections. Each bar represents mean cells ± SEM in 4 high powered fields (HPF) from at least 3 different animals. At each time point, *p* values were calculated using the Mann–Whitney test and represent comparisons between stained cells or nuclei in the injured group with the uninjured group. # *p* = 0.04. * *p* = 0.03. ** *p* = 0.02 compared to uninjured group.

Male C57BL/6 mice aged 18–22 weeks of age underwent left femoral artery wire injury as previously described [31] (Figure 1A). In brief, mice were anesthetized, and a 1.5 cm incision was made directly over the left femoral artery. The right femoral artery served as the uninjured control. The common femoral artery was dissected out along its length. Vascular control was obtained proximally and distally using suture loops. An arteriotomy was performed in a medial muscular arterial branch and a 0.014-inch guide wire was inserted through the arteriotomy into the common femoral artery, passed in and out 3 times, and then held in place for 5 min. Following arterial injury, the guide wire was removed and the muscular branch was ligated. Flow was restored in the femoral artery. At selected time points after surgery, mice were anesthetized, euthanized, and underwent in situ cardiac perfusion with PBS before collection of the injured arterial segment. The location of the injured segment, which is approximately 4 mm in length, was identified using the ligated arterial branch as a distal landmark. Samples used for immunofluorescence were perfusion-fixed with 2% paraformaldehyde prior to collection of the injured arterial segment.

### 2.2. Immunofluorescence and Quantification of Staining

Cryopreserved femoral arteries were prepared as previously described [32]. Five-micron arterial sections were outlined using a hydrophobic barrier pen (ImmEdge; Vector Labs; Burlingame, CA, USA), rehydrated, fixed in 2% paraformaldehyde, and incubated with primary antibodies diluted in IHC-Tek diluent pH 7.4 (IHC-World; Woodstock, MD, USA) for 1 h at room temperature, followed by the appropriate secondary antibody (donkey anti-rat IgG, Alexa Fluor 647; 0.5 μg/mL; Abcam; Cambridge, UK) for 1 h at room temperature. Sections were mounted with ProLong Gold Antifade with DAPI (ThermoFisher Scientific; Waltham, MA, USA). The following primary antibodies were used: rat anti-CD45 (IBL-3/16) (1 mg/mL; Abcam; Cambridge, UK) and rat anti-F4/80 (BM8) (1 mg/mL; Thermo-Fisher Scientific; Waltham, MA, USA).

Digital images were acquired using an Axio Observer D1 Inverted Phase Contrast Fluorescence microscope (Carl Zeiss Microscopy, LLC; Oberkochen, Germany). Positively stained cells and DAPI-stained nuclei in the intimal, medial, and adventitial layers of each high-powered field were separately counted in a blinded fashion by 2 blinded investigtors. At least 4 high-powered fields were sampled per arterial segment from 3 mice.

### 2.3. Preparation of Single-Cell Suspensions

Two femoral arteries were collected and pooled together for each single-cell suspension sample. Each artery was cut into sub-millimeter pieces using microsurgical scissors under a microscope and digested in RPMI buffer supplemented with 0.25 mg/mL of Liberase™ TL (composed of collagenase I/II) (MilliporeSigma; St. Louis, MO, USA), and 1 mg/mL DNase (Roche; Indianapolis, IN, USA) for 1 h at 37 °C with shaking at 200 rpm. Digested samples were sheared 8–10 times using a 21.5-gauge needle attached to a 1 mL syringe and then filtered through a 40 μM nylon mesh filter. Residual tissue was macerated using a rubber syringe plunger. The filtrate was washed with MACS buffer 3 times to generate a single-cell suspension. Single-cell suspensions underwent a 1 min red blood cell lysis step using Pharm Lyse™ buffer (BD Biosciences; Franklin Lakes, NJ, USA) at room temperature, which was halted using ice-cold HBSS. Following RBC lysis, concentration was measured using an automated cell counter (Nexcelom Bioscience; Lawrence, MA, USA).

### 2.4. Immunostaining and Flow Cytometry

A 15-color flow cytometry panel was designed to identify leukocyte subtypes of interest, as shown in Table 1. Antibody concentrations were optimized using single-antibody staining of cell suspensions of murine splenocytes and bone marrow cells with serial antibody titrations to determine the concentration with optimal staining index for each antibody. During these antibody titration steps, splenocyte and bone marrow suspensions were diluted to a cell count of approximately 1.2 × 10^5^ cells/mL to match the expected concentration for a single-cell suspension prepared from two femoral arteries.

Single-cell suspensions were first stained with a fixable live/dead stain to allow for selection of live cells, followed by incubation with Fc Block™ (BD Biosciences), and the staining cocktail for 14 antigens: CD45, MHCII, CD8, CD4, CD19, CD11b, CD11c, CD43, CD64, NK1.1, Ly6G, Ly6C, SiglecF, and Tim4. Finally, the cell suspension was fixed in 2% paraformaldehyde for 8 h at 4 °C. Flow cytometry was performed using a FACSymphony™ A5 Cell Analyzer (BD Biosciences; Franklin Lakes, NJ, USA) and data were captured using BD FACSDiva™ software (BD Biosciences; Franklin Lakes, NJ, USA). The entirety of each sample was acquired and recorded.

Cell populations were identified using a sequential gating strategy (see Results). “Fluorescence minus one” controls were used as necessary. Compensation and gating were performed using FlowJo version 10.8.1 software (BD Biosciences; Franklin Lakes, NJ, USA).

### 2.5. Statistical Analysis

Data are shown as means ± SEM. Differences between means were assessed using Student’s *t*-tests. All analyses were performed using GraphPad Prism, version 9.0 (GraphPad Software; San Diego, CA, USA). *p* < 0.05 was considered statistically significant.

## 3. Results

### 3.1. Immunofluorescence Staining of Femoral Artery Sections

Arterial morphometry of uninjured and injured arteries was assessed by hematoxylin and eosin staining, as shown in Figure 1B,C. As shown in Figure 1C,D, overall leukocyte (CD45) and macrophage (F4/80) infiltration increased starting 1 day after injury, peaked 7 days after injury, and declined between days 7 and 28. Peak inflammatory infiltration occurred prior to the peak neointimal hyperplasia lesion at day 28 (Figure 1C).

### 3.2. Flow Cytometry Gating Strategy for Leukocyte Sub-Populations

**Figure 2:**
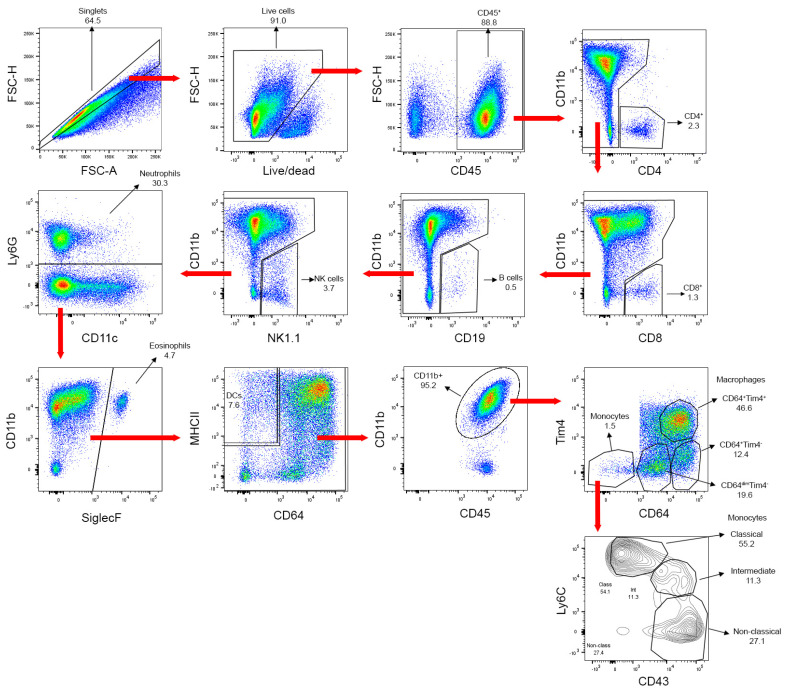
Gating strategy used to identify leukocytes and leukocyte sub-populations in a representative sample with the 15-parameter flow cytometry panel. FSC-H, forward scatter height. FSC-A, forward scatter area. NK cells, natural killer cells. DCs, dendritic cells.

Following routine gating to identify singlets and live cells, a staining mixture containing 14 monoclonal antibodies, each conjugated to different fluorophores, was used for differential identification of leukocyte subpopulations (Table 1) using the gating strategy shown in Figure 2.

### 3.3. Dynamics of Leukocyte Accumulation in Injured Femoral Arteries

**Figure 3:**
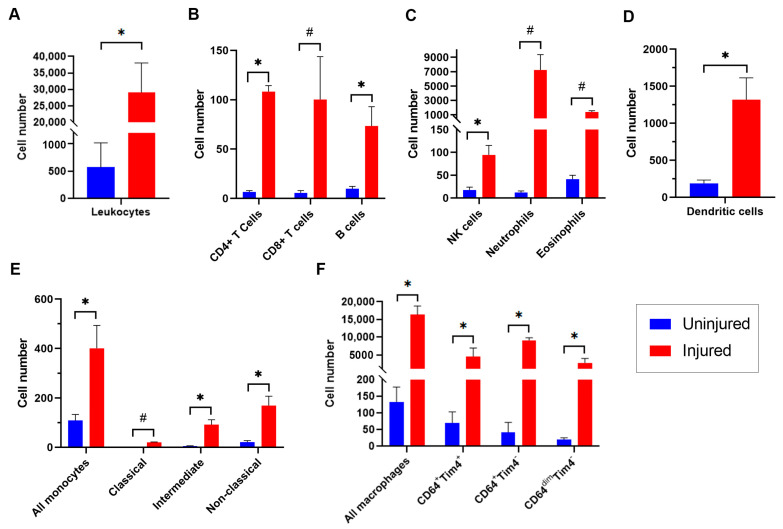
Comparison of leukocytes and leukocyte sub-populations identified using high-parameter flow cytometry in uninjured arteries and in femoral artery samples 3 days after injury. Comparisons are in numbers of live leukocytes (**A**), B and T lymphocytes (**B**), natural killer (NK) cells, neutrophils, and eosinophils (**C**), dendritic cells (**D**), monocytes and monocyte subpopulations (**E**), and macrophages and macrophage subpopulations (**F**). Each bar represents the mean cell number ± SEM of at least 3 samples, each comprising 2 arterial segments. *p* values calculated using Student’s *t*-test. CM, classical monocytes. IM, intermediate monocytes. NC, non-classical monocytes. * *p* = 0.007, # *p* = 0.004.

The accumulation of overall leukocytes and of leukocyte subpopulations was first compared between uninjured arteries and injured arteries at the 3-day timepoint, when an acute inflammatory response is known to occur [7,8,24]. Approximately 32,200 live cells were present in the injured artery samples (each comprising two pooled arteries), of which approximately 29,000 were leukocytes (Figure 3A), representing 58.6-fold more leukocytes per sample than the uninjured artery samples. For all leukocyte subpopulations examined at this time point (lymphocytes, natural killer [NK] cells, neutrophils, eosinophils, dendritic cells, monocytes, and macrophages), the number of cells in each injured artery sample significantly exceeded those in the uninjured artery samples (Figure 3B–F).

**Figure 4:**
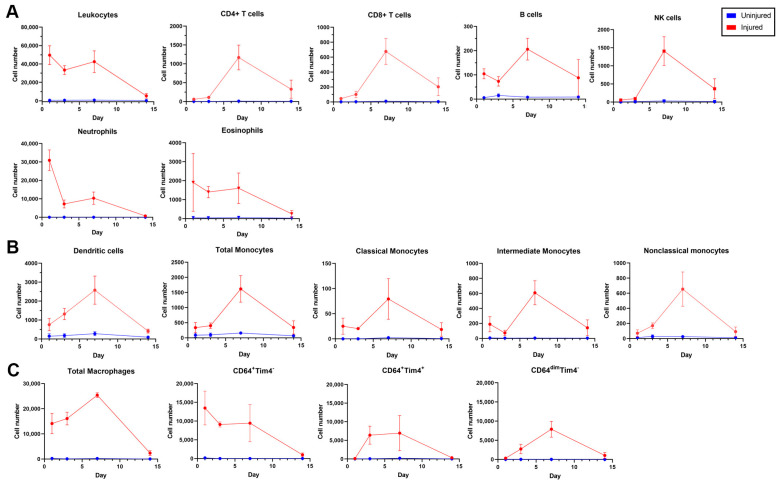
Leukocyte and leukocyte sub-population infiltration in uninjured arteries and at 1, 3, 7, and 14 days after injury using the high-parameter flow cytometry panel. Line graphs show all leukocytes, lymphocytes, neutrophils, natural killer (NK) cells, and eosinophils in injured and uninjured arteries (**A**), dendritic cells, monocytes, and monocyte sub-populations (**B**), and macrophages and macrophage subpopulations (**C**).

The dynamics of leukocyte accumulation from 1–14 days after femoral artery injury are shown in Figure 4. Total leukocyte accumulation was between 29,000 and 49,600 cells in the first 7 days after injury and decreased thereafter, reaching 5400 at 14 days (Figure 4A), a trend which is qualitatively in concordance with the immunofluorescence of arterial sections described above. In uninjured arteries from the same animals, there was an average of 570 live leukocytes/sample, which represented approximately 17.9% of all live cells at each time point. Live leukocyte numbers did not vary over time in the uninjured arteries, suggesting that the trends seen in the injured artery samples do not represent a sample processing artifact (Figure 4A).

Both B and T lymphocytes (CD4+ and CD8+) were sparse and peaked in cell number at 7 days (200 and 1800 per sample, respectively) (Figure 4A). Neutrophils were the most abundant innate cell type in the first 7 days after injury and peaked at 30,900 cells/sample at day 1 before decreasing to 10,300 cells/sample at 7 days and 730 cells/sample at 14 days. NK cells had a similarly rapid accumulation in the first 7 days before declining significantly by 14 days (Figure 4A). Eosinophils remained steadily elevated in the first 7 days before also declining between 7 and 14 days (Figure 4B).

By day 3, monocytes and macrophages represented the largest proportion of all leukocytes, or 18,600 cells/sample, and peaked at 24,500 cells/sample on day 7 (Figure 4B,C), again in concordance with immunofluorescence. Monocytes were further subdivided into fractions of classical (Ly6C^+^CD43^−^), non-classical (Ly6C^−^CD43^+^), and intermediate monocytes (Ly6C^dim^CD43^dim^) (Figure 4B). Three distinct subpopulations of macrophages were identified using CD64 and Tim4. As shown in Figure 4C, the steady increase in CD64^+^Tim4^+^ macrophage accumulation in the first 7 days corresponds to a steady decrease in CD64^+^Tim^−^ macrophages, and both subpopulations had a downward trend after day 7. CD64^+^Tim4^−^ macrophages represented 97.0%, 53.4%, 31.6%, and 30.7% of all macrophages, while CD64^+^Tim4^+^ macrophages represented 0.7%, 26.9%, 37.7%, and 25.0% of all macrophages at 1, 3, 7, and 14 days, respectively.

## 4. Discussion

We performed a 15-parameter flow cytometry study to obtain quantitative and simultaneous characterization of multiple distinct leukocyte subtypes involved in remodeling mouse femoral arteries after wire injury, a commonly used model of neointimal hyperplasia [26,33]. Transluminal mechanical injury and dilation and the ensuing acute inflammatory response incurred by this model closely mimics balloon angioplasty. The major qualitative trends in the flow cytometry findings were validated by immunostaining for CD45 and F4/80, which showed that most leukocytes accumulate within the first 7 days after injury.

As anticipated, we observed an early peak in the neutrophil infiltrate [34], followed by infiltration of monocytes/macrophages [7]. We observed an earlier peak in the monocyte/macrophage infiltrate than previously reported (beginning at day 3 rather than day 7) [7,34], possibly due to differences in mouse age or strain or our technique, which assesses the entire injured segment in comparison to quantitation of staining on selected arterial sections, which may not be representative of the entire injured segment. We also observed a robust inflammatory infiltrate that overall lasted at least 7 days [35], which is also more protracted than previously reported [34].

The finding that CD64^+^Tim4^+^ macrophages steadily increase in number over the first 7 days after arterial injury while CD64^+^Tim4^−^ decrease over the same period has not been previously described. This finding suggests that Tim4 expression was induced by the arterial microenvironment [36] or that Tim4^+^ macrophages trafficked into the artery. Tim4 is a type-I cell-surface glycoprotein that is expressed by professional antigen-presenting cells [37] and may regulate macrophage function [38,39]. Specifically, Tim4 is a negative regulator of nitric oxide production by macrophages [39] and lipopolysaccharide-induced macrophage activation [38], and promotes phagocytosis of apoptotic cells [40,41]. Blockade of Tim4 increases atherosclerosis in mouse models [42] by the prevention of phagocytosis of phosphatidylserine-expressing apoptotic cells and activated T cells by Tim4-expressing cells. Macrophages are the dominant myeloid cells recruited to injured arteries prior to the development of the neointima lesion [43]. Thus, mechanistic studies aimed at understanding specifically how Tim4^+^ macrophages regulate the arterial remodeling process are underway.

A 13-parameter flow cytometry technique was used to characterize total leukocytes, B and T lymphocytes, dendritic cells, monocytes/macrophages, NK cells, and granulocytes in a model of disturbed flow (low and oscillatory shear stress) induced by partial carotid ligation in apolipoprotein-E-deficient mice fed a high-fat diet that leads to atheroma development [44]. This work differs from the current study in its focus on immune cell accumulation from flow disturbance in the context of atherosclerosis rather than restenosis from neointimal hyperplasia after transluminal mechanical injury. This work also did not quantify granulocyte subsets (e.g., neutrophils) or monocyte/macrophage subsets, which are specifically relevant to neointimal hyperplasia.

A high-parameter flow cytometry study allows for the accurate and unbiased quantitation of multiple cell markers in an entire sample simultaneously. Drawbacks are the use of enzymatic digestion and the homogenization of tissue, which could lead to cell death or systematic artifacts in the quantitation of subpopulations. This technique also does not allow for spatial characterization of the cellular infiltrate in the layers of the arterial wall. Nevertheless, we were able to demonstrate in detail the dynamics of leukocyte accumulation that precede neointima formation after arterial injury and to use CD64^+^Tim4^+^ phenotyping to define a discrete macrophage subpopulation that may be important in regulating arterial remodeling.

## 5. Conclusions

In conclusion, we present a multi-parameter flow cytometry method for simultaneous quantitation analysis of live leukocytes and 13 leukocyte subtypes in remodeling segments of mouse femoral arteries after injury, thus allowing for a detailed analysis of the dynamics of inflammatory cell infiltration in the arterial wall. Future studies will be aimed at determining the role of CD64^+^Tim4^+^ macrophages in early arterial remodeling and applying this methodology to studies of fate mapping and cell sorting for transcriptomic or proteomic studies.

## Figures and Tables

**Table 1:** Antibody staining panel and optimal staining concentration for approximately 1 × 10^5^ cells in 50 μL volume.

Fluorophore	Antibody	Marker	Clone	Staining Concentration *	Vendor
BB700	CD8	Cytotoxic T cells	53–6.7	0.5	BD Biosciences (Franklin Lakes, NJ, USA)
BB515	CD43	Monocyte subsets	S7	5	BD Biosciences
AF700	CD4	Helper T cells	RM4-5	3	BD Biosciences
A647	CD64	Monocytes and macrophages	X54-5/7.1	1.25	BD Biosciences
PE/Cy7	MHCII	Professional antigen-presenting cells	M5/114.15.2	3	Invitrogen (Waltham, MA, USA)
PE/CF594	SiglecF	Eosinophils	E50-2440	0.6	BD Biosciences
PE	Tim4	Macrophage subsets	RMT4-54	2.5	BD Biosciences
BV786	NK1.1	Natural killer cells	PK136	1	BD Biosciences
BV711	Ly6G	Neutrophils	1A8	1	BD Biosciences
BV480	Live/Dead	Viability dye	v500	1 μL/2 mL	eBioscience (San Diego, CA, USA)
BV421	Ly6C	Monocytes and macrophages	HK1.4	2.5	BioLegend (San Diego, CA, USA)
BUV805	CD45	Leukocytes	30-F11	0.25	BD Biosciences
BUV737	CD11b	Innate immune cells	M1/70	3	BD Biosciences
BUV563	CD19	B cells	1D3	1.25	BD Biosciences
BUV395	CD11c	Dendritic cells, monocytes, macrophages, granulocytes, NK cells, and subsets of B and T cells	HL3	5	BD Biosciences

* µg/ 50µL unless otherwise indicated.

## Data Availability

The data that were generated during the current study are available from the corresponding author upon reasonable request.
